# Socioecological drivers of burnout – a mixed methods study of military health providers

**DOI:** 10.3389/fpubh.2024.1410825

**Published:** 2024-11-18

**Authors:** Bolanle Olapeju, Ting Dong, Michael Soh, Omar Mushtaq, Hanna Chang, David Riegleman, Paul A. Hemmer, Stephen D. Schwab, Patrice Shanahan, Kimberly Johnson-Boua, Steven J. Durning

**Affiliations:** ^1^Department of Preventive Medicine and Biostatistics, Uniformed Services University of the Health Sciences, Bethesda, MD, United States; ^2^Center for Health Professions Education, Uniformed Services University of the Health Sciences, Bethesda, MD, United States; ^3^375th Medical Group, Flight Operational Medicine Clinic, Scott Air Force Base, IL, United States; ^4^SAUSHEC Medical Education Department, Brooke Army Medical Center, Fort Sam Houston, TX, United States; ^5^Department of Medicine, Uniformed Services University of the Health Sciences, Bethesda, MD, United States; ^6^Alvarez College of Business, University of Texas at San Antonio, San Antonio, TX, United States; ^7^Department of Medical and Clinical Psychology, Uniformed Services University of the Health Sciences, Bethesda, MD, United States

**Keywords:** burnout, military, health provider, socioecological model, wellbeing

## Abstract

**Introduction:**

Health provider burnout is highly prevalent (28–51%) in the US and may contribute to a projected national health provider shortage by 2030. The Socioecological Model (SEM) is a proven conceptual framework used to identify influencing factors and design relevant solutions to improve health outcomes across multiple ecological levels. This study applied the SEM to identify modifiable drivers and solutions of burnout across multiple levels among US Military health providers.

**Methods:**

We conducted a cross-sectional mixed methods study using an online survey (*N* = 129) and in-depth interviews (*N* = 25) of active duty military health providers. Our primary quantitative outcome was self-reported definite, unrelenting, or complete burnout. Our quantitative analysis included chi-square tests of association and bootstrapped multivariable logistic regressions to explore SEM-informed correlates of burnout, controlling for contextual variables. Our qualitative data explored providers individual experience with workplace stress and burnout, providing details on factors influencing burnout at the individual, interpersonal, organizational and military levels. The qualitative data was systematically coded and analyzed using grounded theory.

**Results:**

About two-thirds (63%) of surveyed providers self-reported burnout. Individual-level factors significantly associated with burnout included a positive coping style (AOR = 0.21; 95% CI: 0.08–0.56), perceived control over workload (AOR = 0.17; 95% CI: 0.04–0.66), and satisfaction with the current job (AOR = 0.11; 95% CI: 0.03–0.39). At the organizational level, providers described as overworked (AOR = 10.58; 95% CI: 3.30–33.91) or in hectic or chaotic primary work areas (AOR = 7.54; 95% CI: 2.33–24.38) had higher rates of burnout. At the military level, providers who were happy with their career path and promotion plan (AOR = 0.16; 95% CI: 0.06–0.44) reported lower rates of burnout. The organizational level had the highest cumulative impact of modifiable factors on burnout (AOR: 0.15; 95% CI: 0.06, 0.36). Qualitative interviews corroborated survey findings and highlighted the individual level manifestations of burnout, the role of interpersonal support as mitigators of burnout and the complexity of governmental and military policies impacting provider wellness.

**Discussion:**

Identified factors influencing burnout at various levels may inform potential data-driven interventions to ensure a functional and vibrant US Military health. Data-driven strategies may include opportunities to balance work demands with resources and ability to cope as well as improve positive coping skills, attitudes and experiences related to work.

## 1 Introduction

Burnout is an occupational syndrome resulting from chronic workplace stress that has not been successfully addressed, characterized by (1) feelings of energy depletion or exhaustion; (2) increased mental distance from one’s job, or feelings of negativism or cynicism related to one’s job; and (3) reduced professional efficacy (1–4). With an estimated prevalence of 28 to 51% in the US (5), health provider burnout is of great public health significance linked with and lower job performance (6), suboptimal patient care and satisfaction (7–9), medical errors (9, 10) with substantial costs for caregivers and hospitals (11). In addition, burnout is presumed to contribute to the projected national health provider shortage by 2030 (12–14).

Military health providers are a unique population that provide health care to active duty servicemembers. About half of Army health providers in the Military Health System (MHS) staff reported moderate to high levels of burnout in 2021 (15) while 34 to 43% of Veterans Affairs health system providers experienced burnout (16). In contrast to civilian health providers, military health provider may face additional military stressors such as their rigorous training, intense physical demands (17), workload (18), insufficient rest during military operations (19), post-traumatic stress disorder (20) and increased emotional and mental stress (21).

Addressing burnout is of military significance due to its impact on the quality of health service provision as well as resilience and retention within the military (15, 22, 23). Theoretical models and conceptual frameworks provide valuable insight in the development, implementation and evaluation of intervention to address burnout among health providers. However, only a few frameworks have been explored. The National Academy of Medicine’s Committee on Systems Approaches to Improve Patient Care by Supporting Clinician Well-Being developed a conceptual model of the clinical work system and its relationship to burnout (24), highlighting three interacting system levels—frontline care delivery, health care organization, and external environment. Another model, the Transactional Model of Physician Compassion (25) describes the interconnections between clinician, patient, family, environmental and institutional factors influencing provider compassion, an antecedent to burnout. Most recently, the Office of the U.S. Surgeon General advisory on addressing health worker burnout (26) identified different levels of factors associated with burnout among health workers including societal and cultural, health care system, organizational and workplace. This approach is similar to the Socioecological Model (SEM), a proven conceptual framework used to identify influencing factors and design relevant solutions to improve health outcomes across multiple ecological levels from the micro level (individual) to the macro level (political) (27).

The socioecological model (SEM) recognizes that individuals affect and are affected by a complex range of influences occurring at the individual, interpersonal, community, organization and political levels (28, 29). While it has been positioned as a potentially valuable framework to understand burnout (29, 30) and increase resiliency among frontline workers (31), no study has systematically applied the SEM to understand the correlates of burnout among military health providers. Using the SEM model, modifiable (controllable or changeable) risk or protective factors influencing burnout can be identified (32). The more risk factors a person has across multiple ecological levels, the greater the likelihood of burnout. Understanding the magnitude of modifiable risk or protective factors for burnout across ecological models is crucial in the design of relevant multilevel interventions among military health providers and the MHS.

**Figure 1:**
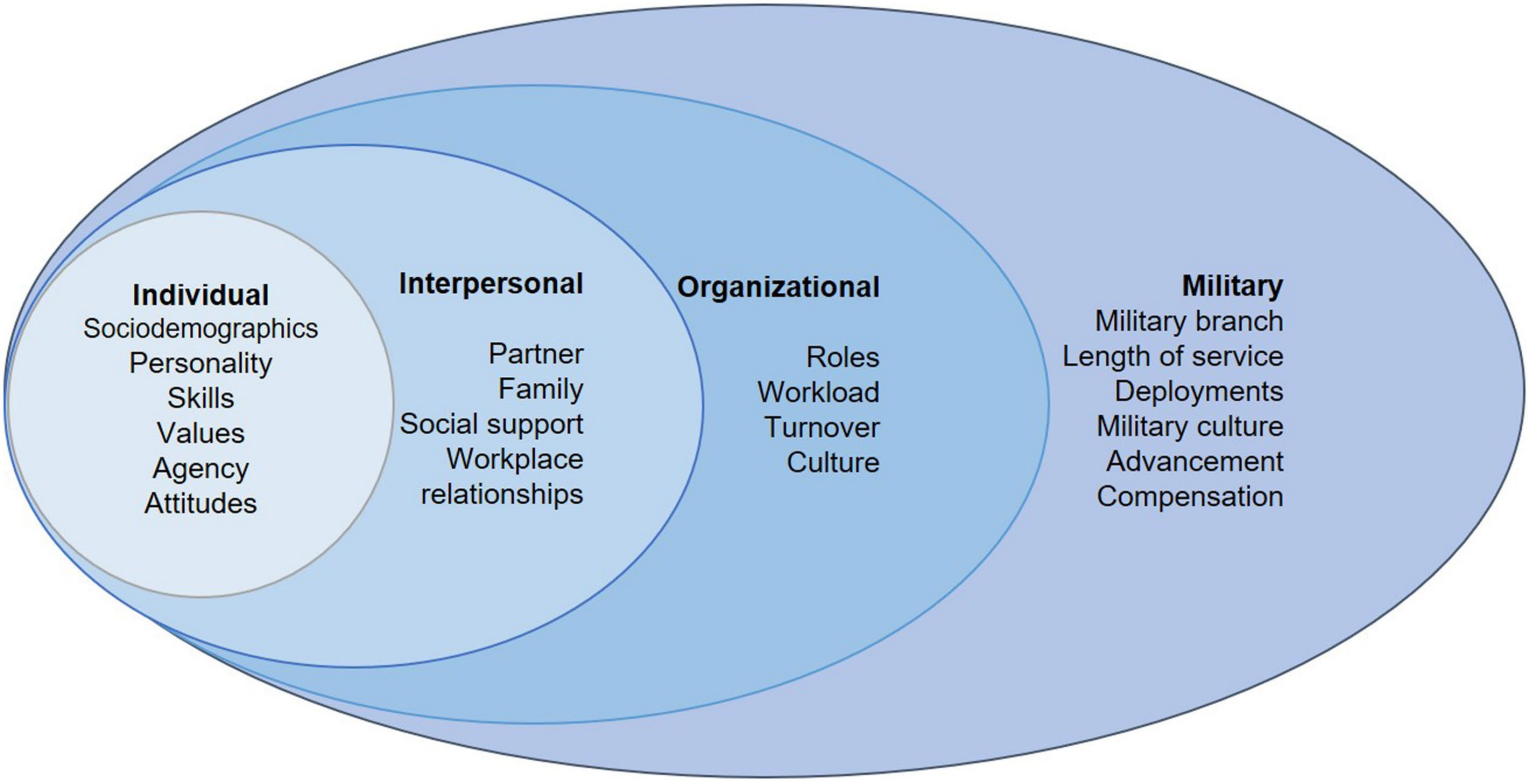
Military provider burnout socioecological model.

The conceptual framework shown in Figure 1 highlights relevant constructs employed by the study. Individual-level constructs included sociodemographic characteristics (such as sex and race/ethnicity), personality, and job-related skills, values, agency and attitudes. The interpersonal level included constructs related to social influence from partners, family, social support and workplace relationships. Organizational constructs related to the delivery of health services and included roles, workload, turnover and culture within the health facility (33). Military level constructs referred to the overarching military community context in which all the military health providers function and included constructs such as military branch, length of service, deployments, military culture and compensation.

While the SEM has not necessarily been explicitly applied to burnout among military health care providers, research suggests multiple factors impact military health care providers’ wellbeing. Individual level factors for burnout among military health providers include age, gender, and rank (34) as well as years in practice, and specialty; with burnout higher among younger providers, urologists, otolaryngologists, and surgeons (1). Interpersonal factors influencing burnout among military providers include marital conflict (35) or spousal depression (36). Organizational factors influencing burnout include lack of leadership support and job autonomy (37), lower compensation (1), workload and patient volume (38) as well as military experience or exposure to war (39). Military-specific factors include deployment status (16) and branch of military service (40). Within the MHS, drivers for healthcare provider burnout also include interrelationships between workload demand, administrative responsibilities, existing resources and healthcare policies (15).

The study explored the SEM level factors influencing burnout among US Military health providers per the following research questions:

1. What modifiable factors are associated with burnout among military health providers?
2. How do military providers navigate their experience with burnout/stress across the SEM levels (individual, interpersonal, organizational and military)?
3. Which SEM levels are most associated with burnout among military health providers?

Answering the above questions will address persisting knowledge gaps on how the SEM model can be applied to military health provider burnout and identify potential strategies that can be implemented to address burnout among military health providers. Such strategies may help mitigate the projected health provider shortage while ensuring high quality health service provision, as well as provider and patient health-related outcomes.

## 2 Methods

### 2.1 Study design and population

The study design is a cross sectional mixed-methods exploratory study of active duty military health providers within the United States. Eligible participants were based on the following inclusion criteria: (i) provided authorized health care within the military health system; (ii) current military service; (iii) aged 18 years and above; and (iv) provided informed consent. There were no exclusion criteria other than failing to meet the study inclusion criteria.

### 2.2 Data collection

#### 2.2.1 Quantitative survey

Participants were recruited via email from a listserv of alumni of Uniformed Services University of the Health Sciences (USU), the United States’ only federal medical school for military service members. The listserv specifically served alumni that were enrolled in a Long-Term Career Outcomes Study, which explored post-graduation outcomes such as burnout and wellbeing among USU alumni. The online survey was sent via email to 605 USU graduates from 2003 to 2018 with email addresses on the listserv. Alumni also forwarded the email to their contacts who were also active-duty military health providers. Data was collected from March to December 2023. The online survey lasted about 30 min on average and collected sociodemographic information, workload, an abbreviated mini-Z burnout survey (41), brief resilience scale (42), typical approach to stress and general coping mechanisms (43), history of burnout among self/colleagues, perceptions related to burnout, and intended retention in military/health service. Of the 605 emails sent, 13 emails bounced back and there were 2 duplicates for a total of 591 unique email recipients. Of the 591 unique email recipients, 56 (9%) attempted the survey. An additional 99 anonymous survey responses were also noted, presumably from the contacts of the email recipients. We are unable to determine the total number of contacts who received a forwarded email and thus cannot assess their response or completion rates. A total of 155 people attempted the questionnaire, 139 people were eligible per the study inclusion criteria and 129 people completed the survey (46 email and 83 anonymous responders). See Supplementary Table 1 which highlights the lack of significant differences among email versus anonymous responders across key demographic variables.

#### 2.2.2 Qualitative in-depth interviews

Some in-depth interview participants were survey respondents who opted in to a follow-up interview, while others were referred to the study by their contacts across specific military treatment facilities based on the principles of snowball sampling (44). The focus of these interviews was to understand health provider context and experiences related to burnout. Interviews lasted roughly for 60 min and were semi-structured to allow for targeted questions and flexibility to explore emerging topics. Interviews were conducted until saturation occurred (*N* = 25). In qualitative research, it is accepted practice to determine non-probabilistic sample sizes by establishing when data saturation occurs, or the point at which nothing new is being learned from the data, assuming a focused domain of inquiry and a certain degree of respondent homogeneity (45). Typically, saturation is achieved at 5 to 30 respondents in a Grounded Theory design (46).

### 2.3 Key variables

#### 2.3.1 Quantitative survey

The quantitative survey questionnaire is presented in Supplemental material 2. Burnout was assessed using the mini-Z burnout survey question: Select the one statement that best describes your experience with burnout. Response categories included: (a) I enjoy my work. I have no symptoms of burnout; (b) I am under stress, and do not always have as much energy as I did, but I do not feel burned out; (c) I am definitely burning out and have one or more symptoms of burnout; (d) The symptoms of burnout that I am experiencing will not go away. I think about work frustrations a lot; (e) I feel completely burned out. I am at the point where I may need to seek help. Response options were also categorized as a binary variable of definite, unrelenting or complete burnout if participants stated the later three options.

Table 1 lists all covariates and their corresponding constructs explored in the quantitative survey and adapted to the individual, interpersonal, organizational and military levels of the SEM per the conceptual framework.

**Table 1:** Study variables.

Variables	Definition/Notes
Burnout	(a) I enjoy my work. I have no symptoms of burnout; (b) I am under stress, and do not always have as much energy as I did, but I do not feel burned out; (c) I am definitely burning out and have one or more symptoms of burnout; (d) The symptoms of burnout that I am experiencing will not go away. I think about work frustrations a lot; (e) I feel completely burned out. I am at the point where I may need to seek help. Burnout yes (responses c–e) versus no (a, b).
Individual	
Sociodemographic	Male versus Female
	Non-Hispanic White versus not
	Physician versus not
	At least 5 years versus not
Personality	Providers described themselves as having a high level of compassion for patients versus not.
	Based on the following Likert scale questions: I″ control my emotions by not expressing them;” “I tend to focus more on the positive aspects of my life than the negative;” “It is hard for me to snap back when something bad happens;” “I usually come through difficult times with little trouble (responses were recoded to reflect positive coping, summed into a composite score ranging 0 to 4 and then categorized as positive (<2) versus not (<2). Scores greater than 2 were considered as a positive coping style).
Skills	Perceived skills and expertise to function in role; Perceived proficiency with electronic health records
Values	Alignment of providers’ professional values with their department leaders.
Agency	Perceived control over their workload
Attitudes	Satisfaction with their current job; If they liked going to work
Interpersonal	
Marital status	Married versus not.
Family	Had child(ren) versus none
Social support	Has at least two confidants for work related stress
Workplace relationships	Felt irritability toward co-workers in the past year; Presence of rewarding relationships with trainees/ colleagues, and Perception that the care team works efficiently together.
Organizational	
Specialty	Surgery, pediatrics/ obstetrics & gynecology, medicine, and other specialty
Workload	Lack of excessive administrative duties; Sufficient time for documentation; Feeling overworked
Turnover	Whether turnover was a significant problem in their practice
Culture	Description of work environment as either hectic or chaotic; Knowledge of organizational efforts to promote wellbeing; Receiving minimal support at work regarding coping with stress; and Reporting four or more positive experiences related to their job in the past year.
Military	
Service branch	Air Force, Army, Navy, Others
Length of service	At least 10 years versus less
Deployments	Any versus none
Culture	Report of military- related stress
Advancement	Happiness with career path and promotion plan
Compensation	Stressed by compensation or financial concerns.

#### 2.3.2 Qualitative in-depth interviews

Interviews focused on specific aspects of military health care providers’ experience burnout, such as their most recent experience with burnout, interpersonal relationships between themselves, families, colleagues, and supervisors, their relationship to organizational factors as well as their work performance, relevant policies that interact with their experience as health care providers.

### 2.4 Analytical methods

#### 2.4.1 Quantitative survey

Stata version 18 (Stata Corporation, College Station, TX, USA) was used for data management and analysis. Missing data was negligible (< 1%) for all variables except military health provider race/ethnicity (6%) and specialty (4%). Analyses reclassified missing data using simple imputation (assigned to the most frequent category). Cross tabulations were used to estimate frequencies, percentages and standard errors and chi-square tests of association of study variables by service branch as well as burnout status. Bootstrapped multivariable logistic regression models using 1,000 replications were employed to explore specific modifiable factors as well as the cumulative impact of modifiable factors across the SEM levels. The study employed bootstrapped models to enable more robust statistical inferences given the relatively more sample size. In the first model, the main covariates included the modifiable factors that were significantly associated with burnout from the chi-square tests of association. In the second model, the main covariate was a standardized score for all the modifiable factors in each of the SEM levels. Modifiable factors were recoded to be protective as needed in order to ensure uniform directionality. Both models adjusted for non-modifiable characteristics considered to be key contextual factors including military branch, sex, race/ethnicity, deployment status and length of military service.

#### 2.4.2 Qualitative interviews

The data was systematically coded and analyzed via grounded theory which enables respondents explain how they make meaning of their realities and how these meanings influence behavior (47). Memo analysis was used to describe current data, identify patterns, and identify possible areas of exploration. Open codes were systematically assigned through a line-by-line schema, and subsequently, axial codes for coding contextual information as well as selective codes for specific phenomena. These codes were inductively congealed into themes. Finally, all responses were collected to ensure confidentiality and anonymity of respondents. As such, pseudonyms were issued to ensure responses were anonymous.

### 2.5 Ethical considerations

This study was reviewed and determined to be exempt research by the USU Human Research Protection Program Office (HRPPO), USU IRB Protocol Reference Number 22–16117. All participants provided written informed consent in the survey. All participants confirmed that they reviewed the study information and consented to be enrolled in the study. Participants’ confidentiality was ensured throughout the study and only identifying information was used.

## 3 Results

### 3.1 Description of study population

As shown in Table 2, of the 129 military health providers who completed the survey, over half identified as male (*n* = 75, 58%) while a majority were physicians (*n* = 110, 95%), married (*n* = 111, 86%), White (*n* = 104, 81%) or had children (*n* = 92, 71%). Represented specialties included surgery, pediatrics, obstetrics and gynecology, internal medicine and others. Eighty seven percent of survey participants reported more than 10 years of military service (*n* = 101) and slightly over half had been deployed at least once (*n* = 74, 57%).

**Table 2:** Description of study population by service branch.

Quantitative survey (*N* = 129)						
Characteristic (*N* (%))	Air Force 57 (44%)	Army 38 (30%)	Navy 34 (26%)	Total 129 (100%)	Chi square 𝛘^2^	*p*-value
Male sex	27 (47%)	28 (74%)	20 (59%)	75 (58%)	6.497	**0.039**
White	46 (81%)	29 (76%)	29 (85%)	104 (81%)	0.926	0.629
Physician	51 (89%)	37 (97%)	34 (100%)	122 (95%)	5.418	0.067
Specialty						
Surgery	2 (4%)	11 (29%)	6 (18%)	19 (15%)	12.061	**0.002**
Pediatrics/ Obstetrics Gynecology	10 (18%)	8 (21%)	3 (9%)	21 (16%)	2.089	0.352
Medicine	27 (47%)	9 (24%)	12 (35%)	48 (37%)	5.547	0.062
Other^1^	18 (32%)	10 (26%)	13 (38%)	41 (32%)	1.178	0.555
Practicing for over 5 years	23 (40%)	13 (34%)	15 (44%)	51 (40%)	0.765	0.682
More than 10 years of service	38 (67%)	34 (89%)	29 (85%)	101 (87%)	8.310	**0.016**
Deployed at least once	28 (49%)	23 (61%)	23 (68%)	74 (57%)	3.208	0.201
Married	52 (91%)	32 (84%)	27 (79%)	111 (86%)	2.628	0.269
Has children	40 (70%)	30 (79%)	22 (65%)	92 (71%)	1.844	0.398
Qualitative in-depth interviews (*N* = 25)						
Characteristic (*N* (%))	Army 5 (20%)	Navy 5 (20%)	Air Force 15 (60%)	Total 25 (100%)	Not applicable	
Male	5 (100%)	4 (80%)	6 (40%)	15 (60%)		
Female	0	1 (20%)	9 (60%)	10 (40%)		

1 Other includes anesthesia, primary care, flight-, occupational-, and operational- medicine, pathology, radiology. The bolded *p*-values were to highlight statistically significant *p*-values, less than 0.05.

Of the 25 in-depth interview participants, five respondents were in the Army and Navy each while 15 respondents were in the Air Force. Most participants (60%) were male.

### 3.2 Individual and interpersonal level

Table 3 highlights the quantitative association between burnout and individual and interpersonal level factors. For factors at the individual level, four survey items showed statistically significant associations with the outcome of burnout – *Positive coping style* (*χ*^2^ = 12.96, *p* < 0.01), *Perceived control over workload* (*χ*^2^ = 7.77, *p* < 0.01), *Satisfied with current job* (*χ*^2^ = 23.37, *p* < 0.001), and *Likes going to work* (*χ*^2^ = 27.16, *p* < 0.001). The respondents who answered yes to any of these four items were less likely to experience burnout. At the interpersonal level, one factor turned out to have statistically significant relationships with burnout–respondents who noted irritability with their co-workers in the past year were more likely to experience burnout.

**Table 3:** Individual and interpersonal factors influencing burnout.

Factor (*N* (%))	Constructs	No burnout 48 (37%)	Burnout present 81 (63%)	Total 129 (100%)	Chi square 𝛘^2^	*p*-value
Individual factors						
Male sex	Sociodemographics	28 (58%)	47 (58%)	75 (58%)	0.001	0.973
White		37 (77%)	67 (83%)	104 (81%)	0.612	0.434
Physician		45 (94%)	77 (95%)	122 (95%)	0.101	0.751
Practicing at least 5 years		19 (40%)	32 (40%)	51 (40%)	0.000	0.993
Describes themselves as compassionate toward patients	Personality	32 (67%)	44 (54%)	76 (59%)	1.898	0.168
Positive coping style		27 (56%)	20 (25%)	47 (36%)	12.961	0.001
Perceived skills and expertise to function in role	Skills	46 (96%)	77 (95%)	123 (95%)	0.041	0.841
Perceived proficiency with EHR		19 (40%)	37 (46%)	56 (43%)	0.456	0.500
Professional values are well aligned with department leaders	Values	38 (79%)	52 (64%)	90 (70%)	3.202	0.074
Perceived control over workload	Agency	12 (25%)	6 (7%)	18 (14%)	7.769	0.005
Satisfied with current job	Attitudes	41 (85%)	34 (42%)	75 (58%)	23.371	<0.001
Likes going to work		41 (85%)	31 (38%)	72 (56%)	27.163	<0.001
Interpersonal factors						
Married	Partner	42 (88%)	69 (85%)	111 (86%)	0.135	0.714
Has children	Family	35 (73%)	57 (70%)	92 (71%)	0.096	0.757
Has confidants regarding work struggles	Social support	46 (96%)	77 (95%)	83 (71%)	0.044	0.834
Irritable toward co-workers in past year	Workplace relationships	21 (44%)	50 (62%)	71 (55%)	3.937	0.047
Notes rewarding relationships with trainees/ colleagues		39 (81%)	53 (65%)	92 (71%)	3.687	0.055
Perceives care team works efficiently together		22 (46%)	32 (40%)	54 (42%)	0.496	0.481

**Figure 2:**
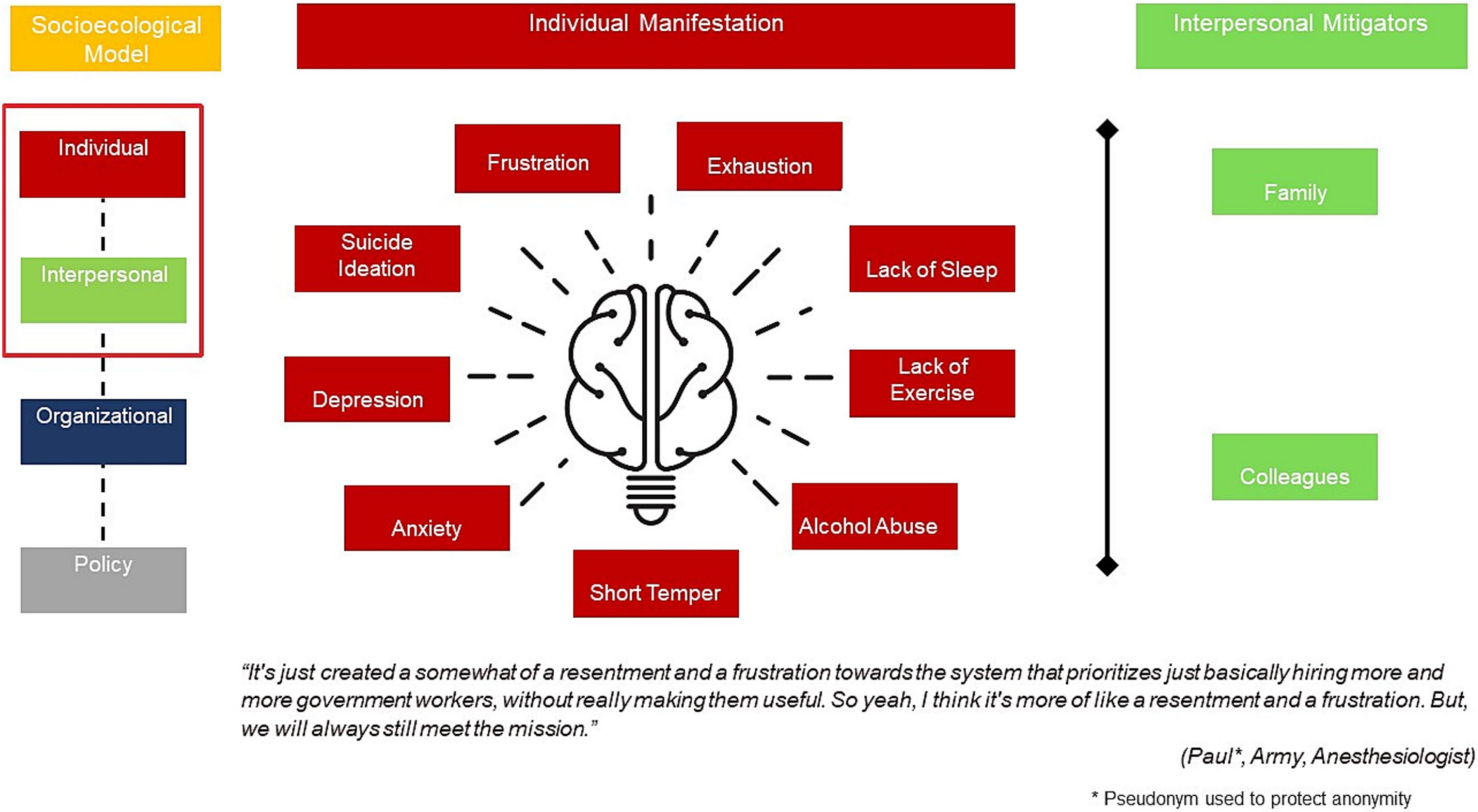
Individual manifestation and interpersonal mitigators of burnout.

In-depth interview respondents shared their experiences with burnout including the different ways burnout manifested as well as the role of their interpersonal relationships. As summarized in Figure 2, military providers typically experienced frustration with their inability to perform their roles as clinicians due to bureaucracy. Due to increased workloads, they frequently encountered exhaustion, lack of sleep and lack of exercise. This then resulted in alcohol abuse as well as emotional manifestations of short temper, anxiety and in other cases depression and suicide ideation.

On the other hand, interpersonal connections from family and colleagues were noted as mitigators of burnout. Respondents confided in their spouses, family members and work colleagues and shared their experiences with stressors and frustrations.

### 3.3 Organizational level

Among the factors at the organizational level (Table 4), most of them had statistically significant associations with burnout, including *Noted sufficient time for documentation* (*χ*^2^ = 9.34, *p* < 0.01), *Describes themselves as overworked* (*χ*^2^ = 26.02, *p* < 0.001), *Noted turnover as a significant problem in practice* (*χ*^2^ = 5.00, *p* < 0.05), *Describes primary work area as hectic or chaotic* (*χ*^2^ = 18.91, *p* < 0.001), *Knew organizational mechanisms to promote wellbeing* (*χ*^2^ = 5.71, *p* < 0.05), *Perceived minimal support at work regarding coping with stress* (*χ*^2^ = 27.72, *p* < 0.001), and *Noted four or more positive experiences in past year* (*χ*^2^ = 12.77, *p* < 0.001). Providers who described themselves as overworked, who or noted turnover as an issue and who described their work area as hectic or chaotic were more likely to be burned out. Conversely, those who noted sufficient time for documentation, knew organizational wellbeing mechanisms, perceived minimal support at work and had positive experiences were less likely to be burned out.

**Table 4:** Organizational level factors influencing burnout.

Factor (*N* (%))	Constructs	No burnout 48 (37%)	Burnout present 81 (63%)	Total 129 (100%)	Chi square 𝛘^2^	*p* value
Specialty	Specialty					
Surgery		9 (19%)	10 (12%)	19 (15%)	0.984	0.321
Pediatrics/ OBGYN		6 (12%)	15 (19%)	21 (16%)	0.801	0.371
Medicine		15 (31%)	33 (41%)	48 (37%)	1.162	0.281
Other		18 (38%)	23 (28%)	41 (32%)	1.152	0.283
Noted excessive administrative tasks	Workload	39 (81%)	73 (90%)	112 (87%)	2.074	0.150
Noted sufficient time for documentation		12 (25%)	5 (6%)	17 (13%)	9.337	0.002
Describes themselves as overworked		8 (17%)	51 (63%)	59 (46%)	26.029	<0.001
Noted turnover as a significant problem in practice	Turnover	38 (79%)	75 (93%)	113 (88%)	5.000	0.025
Describes primary work area as hectic or chaotic		6 (12%)	41 (51%)	47 (36%)	18.908	<0.001
Knew organizational mechanisms to promote wellbeing	Culture	30 (62%)	33 (41%)	63 (49%)	5.711	0.017
Perceived minimal support at work regarding coping with stress		21 (44%)	72 (89%)	93 (72%)	30.523	<0.001
Noted four or more positive experiences^a^ in past year		35 (73%)	31 (38%)	66 (51%)	14.478	<0.001

OB/GYN, Obstetrics/gynecology.

a Options included: Professional fulfillment; Gratitude from patients; Rewarding relationships with trainees/ colleagues; Validation of hard work/efforts; Satisfaction with performance; Confidence in clinical ability; Intellectual stimulation; Demonstration of good leadership/management skills; Professional recognition.

**Figure 3:**
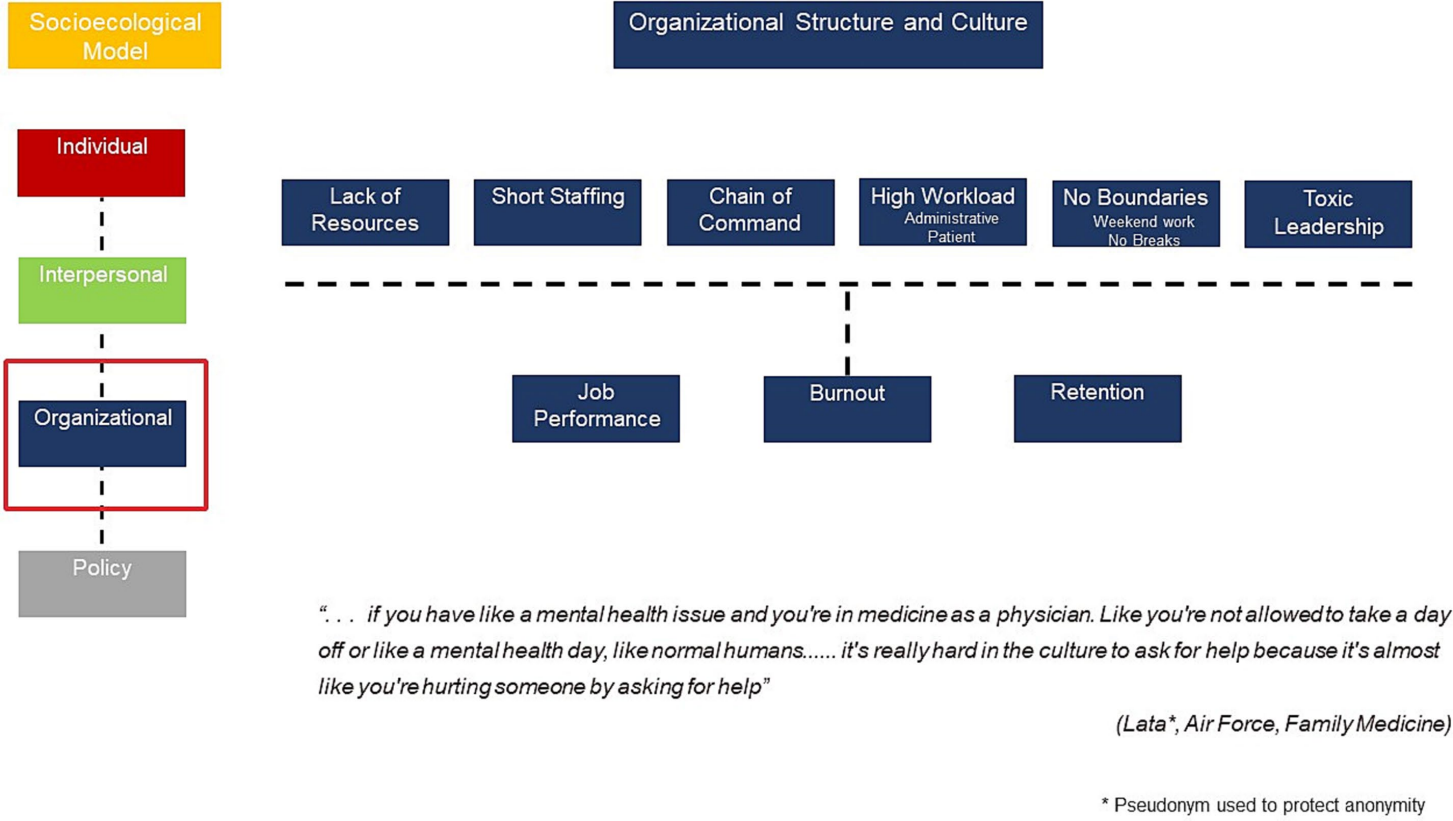
Organizational culture and structure impacting burnout.

In-depth interview respondents articulated several organizational factors impacting their burnout experience as shown in Figure 3. Structural factors included short staffing (particularly clinicians and support staff), lack of resources and the additional pressures of combining clinical care with communicating with the relevant chains of command. Cultural factors related to the norm that very high workloads involving both administrative and clinical tasks were acceptable. In addition, respondents noted that there were no work-life boundaries as they were constantly working late into the night and over the weekend. Several respondents complained that they not able to take breaks or vacation from work easily. There was also reports of toxic leadership as well as a lack of ability to institute helpful changes the work setting to improve provider wellbeing.

### 3.4 Military level

In contrast, military factors under investigation were mostly not influential (Table 5). Only one item had a statistically significant relationship with burnout – *Happy with career path and promotion plan* (*χ*^2^ = 18.92, *p* < 0.001).

**Table 5:** Military level factors influencing burnout.

Factor (*N* (%))	Constructs	No burnout 48 (37%)	Burnout present 81 (63%)	Total 129 (100%)	Chi square 𝛘^2^	*p* value
Air force	Military branch	18 (38%)	39 (48%)	57 (44%)	1.386	0.239
Army		14 (29%)	24 (30%)	38 (30%)	0.003	0.956
Navy		16 (33%)	18 (22%)	34 (26%)	1.912	0.166
More than 10 years of service	Length of service	34 (71%)	67 (83%)	101 (78%)	2.504	0.114
Deployed at least once	Deployments	27 (56%)	47 (58%)	74 (57%)	0.039	0.844
Stressed by military-related demands	Military culture	33 (69%)	53 (65%)	86 (67%)	0.149	0.699
Happy with career path and promotion plan	Advancement	35 (73%)	27 (33%)	62 (48%)	18.918	<0.001
Stressed by compensation or financial concerns	Compensation	12 (25%)	29 (36%)	41 (32%)	1.622	0.203

**Figure 4:**
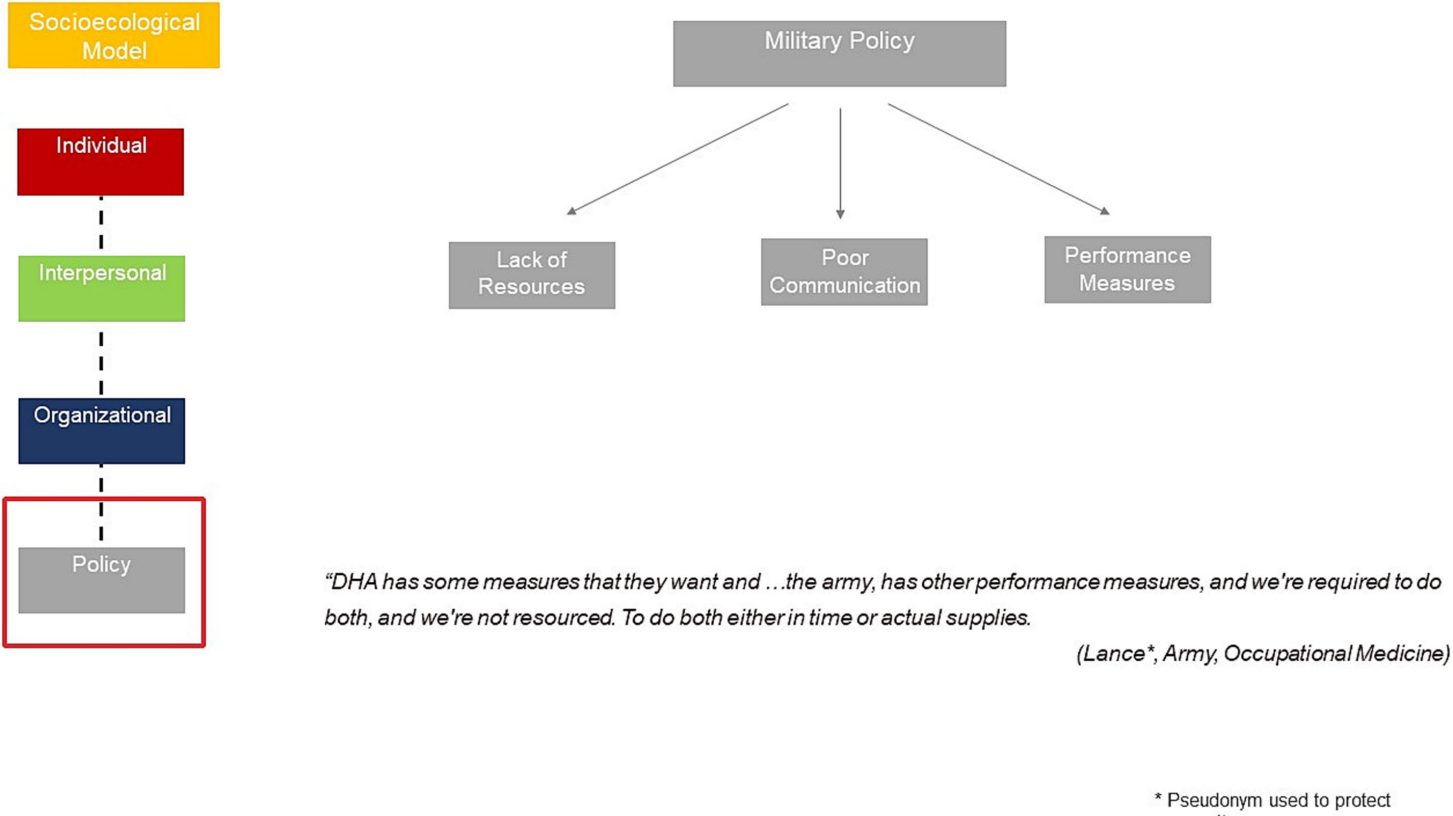
Military policies influencing burnout.

In the in-depth interviews, respondents shared sentiments regarding policy considerations impacting their wellbeing and burnout from the Defense Health Agency (DHA)- the joint integrated agency that oversee the delivery of health care services for the US Army, Navy and Air Force. Examples were shared of Department Health Agency policies that reduced budgetary allocations, leading to reduced or lower quality staff and declining resources. In addition, some of the DHA policies appeared to incomplete and purposely vague, leaving a lot of interpretation. Finally, respondents discussed the duplicative administrative requirements and performance measures from both DHA and their service branches (Figure 4).

### 3.5 Adjusted odds of burnout

Table 6 demonstrated the results of multiple factor logistic regression analysis on a series of select items. The following items were statistically significantly associated with burnout – *Positive coping style* (OR = 0.26, adjusted OR = 0.21), *Perceived control over workload* (OR = 0.24, adjusted OR = 0.17), *Satisfied with current job* (OR = 0.12, adjusted OR = 0.11), *Likes going to work* (OR = 0.11, adjusted OR = 0.10), *Notes sufficient time for documentation* (OR = 0.20, adjusted OR = 0.17), *Describes themselves as overworked* (OR = 8.50, adjusted OR = 10.58), *Describes primary work area as hectic or chaotic* (OR = 7.17, adjusted OR = 7.54), *Knows organizational mechanisms to promote wellbeing* (OR = 0.40, adjusted OR = 0.41), *Perceived minimal support at work regarding coping with stress* (OR = 0.10, adjusted OR = 0.09), *Noted four or more positive experiences in past year* (OR = 0.23, adjusted OR = 0.23), and *Happy with career path and promotion plan* (OR = 0.19, adjusted OR = 0.16). The results of the logistic regression analysis were consistent with those of the chi-square test of independence.

**Table 6:** Adjusted odds ratios of burnout by select SEM factors.

Bootstrapped estimates and 95% confidence intervals (CI) of burnout				
Factor	OR	95% CI	AOR^a^	95% CI
Individual				
Positive coping style	0.26***	0.12–0.54	0.21**	0.08–0.56
Perceived control over workload	0.24*	0.07–0.77	0.17*	0.04–0.66
Satisfied with current job	0.12***	0.04–0.34	0.11***	0.03–0.39
Likes going to work	0.11***	0.04–0.28	0.10***	0.03–0.32
Interpersonal				
Irritable toward co-workers in past year	2.07	0.99–4.34	2.03	0.85–4.82
Organizational				
Noted sufficient time for documentation	0.20**	0.06–0.64	0.17*	0.03–0.96
Describes themselves as overworked	8.50***	3.36–21.50	10.58***	3.30–33.91
Noted turnover as a significant problem in practice	0.30	0.09–1.05	0.34	0.10–1.24
Describes primary work area as hectic or chaotic	7.17***	2.47–20.85	7.54***	2.33–24.38
Knows organizational mechanisms to promote wellbeing	0.41*	0.19–0.89	0.40*	0.17–0.95
Perceived minimal support at work regarding coping with stress	0.10***	0.04–0.26	0.09***	0.02–0.31
Noted four or more positive experiences^b^ in past year	0.23***	0.10–0.51	0.23**	0.09–0.58
Military				
Happy with career path and promotion plan	0.19***	0.08–0.41	0.16***	0.06–0.44

* *p* < 0.05, ** *p* < 0.01,*** *p* < 0.001.

a Adjusted for military branch, sex, race, deployment status and length of military service.

b Experiences included: Professional fulfillment; Gratitude from patients; Rewarding relationships with trainees/ colleagues; Validation of hard work/efforts; Satisfaction with performance; Confidence in clinical ability; Intellectual stimulation; Demonstration of good leadership/management skills; Professional recognition.

**Figure 5:**
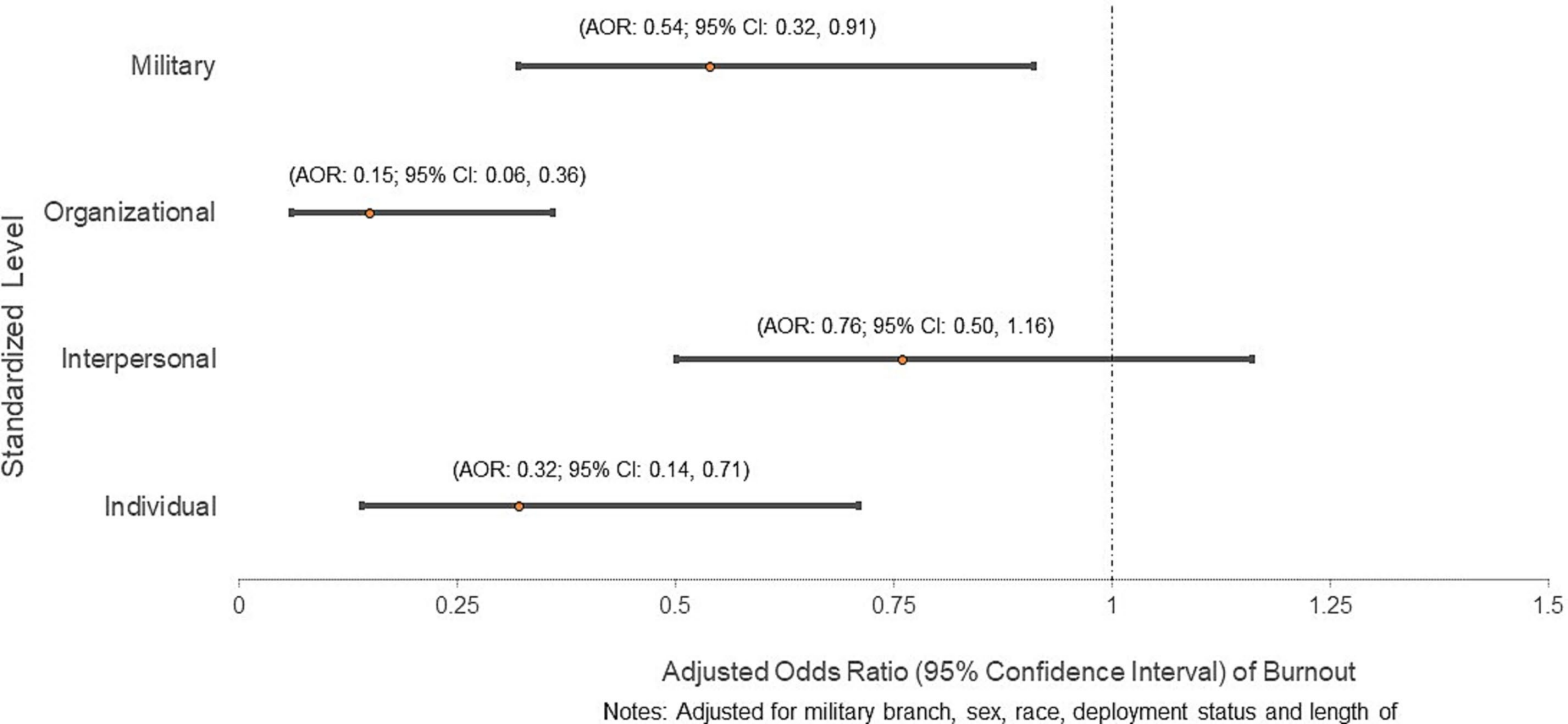
Adjusted odds ratio of burnout by standardized socioecological level scores.

Figure 5 presents the relationship between burnout and the cumulative impact of modifiable factors across the SEM levels. The cumulative impact of modifiable factors is presented as a standardized score of all modifiable factors. Having an additional modifiable individual level factor significantly reduced the odds of burnout (AOR: 0.32; 95% CI: 0.14, 0.71). Similarly, additional modifiable factors at the organizational (AOR: 0.15; 95% CI: 0.06, 0.36) and military (AOR: 0.54; 95% CI: 0.32, 0.91) level reduced the odds of burnout. On the other hand, interpersonal factors were not significantly associated with burnout.

## 4 Discussion

This study explored modifiable factors and socioecological levels associated with burnout among the unique population of military health providers. Study findings suggest that burnout is influenced by several modifiable factors across the SEM and that the cumulative impacts of modifiable factors were most significant at the individual, organizational and military level. At the individual and interpersonal level, beyond possessing a positive coping style, a provider’s sense of agency, their attitudes toward their work and the quality of their professional relationships seemed to play a role in mitigating burnout. At the organizational/military level, workplace culture and workload seemed to play a primary role in contributing to, and mitigating, burnout. For example, feeling overworked and working in chaotic spaces were workload-related correlates of feeling burnt out. However, positive experiences such as gratitude from patients, intellectual stimulation, or professional recognition - all of which contribute to being satisfied with career and or promotion plans - were seen as mitigators of burnout. In-depth interviews aligned with the survey findings, highlighting the individual level manifestations of burnout, the role of interpersonal support as mitigators of burnout and the complexity of governmental and military policies impacting provider wellness. Overall, study findings corroborate recent research findings from larger samples of Army (15) and civilian health providers (1, 33) where concerns about workload, low job satisfaction, administrative demands; and organizational commitment to a healthy workplace were significantly associated with burnout (15).

Overall, the Socioecological Model appears to be well suited for exploring burnout among military health providers as evidenced by its core principles (27). The SEM opines that factors work across multiple levels to influence behavior and that addressing behaviors at multiple levels is most effective in supporting behavior change. The SEM also employs a reciprocal determinism perspective which explores the interaction of the person, behavior, and the environment should be taken into account in the design of relevant interventions. Study findings reiterate this position and suggest that intervening at the individual-, organizational- and military-level can improve military provider burnout. Interventions targeting multiple levels (27, 48) are more likely to promote wider-reaching and longer-term change compared to interventions that focus on just one level (49). Adopting such systems thinking approaches help to understand burnout more holistically, thus identifying specific levers to mitigate burnout (24, 50).

Given the study findings where having a positive coping style was associated with reduced burnout rates, opportunities to improve individual-level factors and positive coping skills among health providers can start in training institutions, where the seeds of well-being can be planted early. These include but are not limited to increased transparency related to burnout, promoting a culture of well-being, providing relevant socio-emotional support, leadership and management training during medical training. At the organizational level, tailored mechanisms should be data driven and practical. For example, providers who noted insufficient time for documentation, described themselves as overworked, or noted significant turnover in their practice were more likely to be burned out. This suggests user-friendly clinical decision support tools (51), virtual mental health care services providing real time support for overburdened workers where they are and on their schedule (13) can help address burnout among providers. Finally, per our study findings, providers perceived minimal support at work and knew few mechanisms to promote wellbeing. Thus, social and behavior change communication interventions should aim to inspire health providers by increasing the number of positive work-related experiences and influencing their personal attitudes. These may include sharing patient gratitude, publicly recognizing providers’ specific contributions, and re-affirming their value to the Military Health System and mission.

This study offers specific implications for clinicians and policy makers for consideration once corroborated by larger scale studies. Per findings on overworked providers being more burned out, opportunities to balance clinicians’ work demands and ability to cope should be implemented as *a priori*ty. An example is task shifting, a rational redistribution of tasks among health workforce teams where specific tasks are moved, where appropriate, from highly qualified health workers to health workers with shorter training and fewer qualifications in order to make more efficient use of the available human resources for health (52). Research suggests that tasks, particularly administrative or documentation tasks, can be shifted from health workers to patients and their caregivers, to machines, and to other health workers (53). This is important given the study finding of an association between insufficient time for documentation and burnout. In addition to hiring additional staff where needed, structure and order may be instituted to the degree possible by clearly delineating roles and responsibilities, clarifying and managing expectations, timelines and priorities to align desired outcomes with contextual realities. This might help improve health provider satisfaction with their workload, career plans and promotion trajectory, identified factors linked with burnout from this study.

This study has implications for Department of Defense (DoD) interests, including improved patient satisfaction, higher physician retention rates, better morale, improved resilience and recruitment within the military. Furthermore, the Military Health System 2024–2029 strategy seeks to restore the well-being of health care personnel so that they are ready to provide the best care and the best support for those who go into harm’s way (54). Reducing health provider burnout will also reduce its negative consequences on patient care, medical readiness sustainment, the physician workforce, and healthcare system costs, in addition to providers’ own care and safety. The identified multi-level factors associated with burnout may facilitate the direction or prioritization of ongoing continuous process improvement projects in the military health system and optimization of the proposed multifaceted interventions. The aforementioned potential burnout solutions should be adequately researched and piloted in military treatment facilities before being rolled out at scale. A holistic approach for addressing burnout among military health providers calls for major improvements in clinical work and learning environments in all settings, and for all disciplines to prevent and mitigate clinician burnout and foster professional well-being for the overall health of clinicians, patients, and the nation (24).

This study boasts several strengths and limitations. To the best of our knowledge, this is the first study to systematically apply the well-proven SEM to fill understand burnout among military health providers and inform the design of interventions. The categorizations of constructs in the SEM model was based on *a priori* knowledge and best judgment, as there is no consistent definition in the literature. Next our statistical analyses accounted for limitations with sample size to ensure robust inferences. We however acknowledge that the cross-sectional study design limits the ability to make any causal conclusions on burnout causality. We are unable to determine the total number of email surveys sent and thus cannot assess their response or completion rates. Thus, the study’s indeterminate response rates and non-random sampling hamper any attempt at generalizability. Finally, the study did not exclude provides with any psychological conditions and the data was not linked to relevant provider comorbidities or outcomes such as depression, anxiety or PTSD across the Department of Defense Military Health System. Due to these limitations, this research is positioned as an exploratory study that will benefit from prospective larger scale studies that address the aforementioned gaps.

There are some research implications of our study findings that can inform future investigation. Given the cross-sectional nature, relatively small sample size, sampling considerations resulting in unclear denominator and few interpersonal factors explored in the quantitative survey, prospective large-scale, generalizable quantitative research should identify more objective interpersonal level factors and explore their relationship with burnout. In-depth interviews can be complemented with focus groups studies that explore group level dynamics and differences related to military health providers’ workload and the concept of being overworked. Such research should seek to better uncover their specific tasks and what they mean by feeling overworked. This may include but is not limited to seeing too many patients, appointment and patient interaction at clinics being too short, clinical and/or administrative duties without sufficient time offsets, lack of sufficient administrative support such that providers/physicians are forced to take on other non-physician duties (e.g., vital signs, completing prior authorization paperwork, etc.).

## 5 Conclusion

In conclusion, amidst a backdrop of rising health provider burnout in the US, this study applied the SEM to the context of military health provider burnout and identified individual, organizational and military level factors influencing burnout among US Military health providers. Potential data driven interventions to ensure a functional and vibrant health provider force may include opportunities to improve positive coping skills, increase favorable attitudes related to work and balance work demands with ability to cope.

## Data Availability

Data are available from the authors upon reasonable request and with permission of DOD. Requests to access these datasets should be directed to the corresponding author.

## Supplementary material

Supplementary Table 1 Description of Email versus Anonymous Survey Participants.

Supplementary Table 2 Military Health Provider Survey Questionnaire.
