# Supplementary material for: Socioecological drivers of burnout – a mixed methods study of military health providers
Source: Front Public Health. 2024 Nov 18;12:1410825. doi: 10.3389/fpubh.2024.1410825 (PMC11609726; doi:10.3389/fpubh.2024.1410825)
Supplement: Supplementary Table 1 — Description of Email versus Anonymous Survey Participants. [file Table_1.docx]

Socioecological Drivers and Solutions of Burnout - a Mixed Methods Study of Military Health Providers

Supplementary Material

## Supplemental Table 1: Description of Email versus Anonymous Survey Participants

| **Characteristic (N (%))** | **Anonymous 83 (64%)** | **Email**  **46 (36%)** | **Total**  **129 (100%)** | **Chi square 𝛘^2^** | **p-value** |
| --- | --- | --- | --- | --- | --- |
| Male sex | 44 (53%) | 31 (67%) | 75(58%) | 2.515 | 0.113 |
| White | 64 (77%) | 40 (87%) | 104(81%) | 1.837 | 0.175 |
| Army | 24 (29%) | 14 (30%) | 38 (30%) | 0.033 | 0.856 |
| Physician | 76 (92%) | 46 (100%) | 122(95%) | 4.102 | 0.043 |
| Practicing for over five years | 31 (37%) | 20 (43%) | 51(40%) | 0.465 | 0.495 |
| More than 10 years of service | 61 (73%) | 40 (87%) | 101(87%) | 3.157 | 0.076 |
| Deployed at least once | 46 (55%) | 28 (61%) | 74(57%) | 0.359 | 0.549 |
| Married | 71 (86%) | 40 (87%) | 111 (86%) | 0.049 | 0.824 |
| Has children | 57 (69%) | 35 (76%) | 92(71%) | 0.795 | 0.373 |
