# Supplementary material for: Socioecological drivers of burnout – a mixed methods study of military health providers
Source: Front Public Health. 2024 Nov 18;12:1410825. doi: 10.3389/fpubh.2024.1410825 (PMC11609726; doi:10.3389/fpubh.2024.1410825)
Supplement: Supplementary Table 2 — Military Health Provider Survey Questionnaire. [file Supplementary_file_2.docx]

Socioecological Drivers and Solutions of Burnout - a Mixed Methods Study of Military Health Providers

Supplementary Material

## Military Health Provider Survey Questionnaire

| **N.** | **Question** | **Responses** |
| --- | --- | --- |
| Section 1: We’d like to know a little bit about you | | |
| 1 | Which of these best describes your race/ethnicity (select all that apply) | Asian 1  Black or African American 2  Hispanic 3  White 4  Other 8  Don’t wish to answer 9 |
| 2 | What is your sex | Male 1  Female 2  Other 8  Don’t wish to answer 9 |
| 3 | What branch of service are you currently in? | Air Force 1  Army 2  Marine Corps 3  Navy 4  Coast Guard 5  Other(specify) 8  Don’t wish to answer 9 |
| 4 | How long have you served in the military | \|___\|___\| years  Don’t wish to answer 9 |
| 5 | Marital status | Not married 0  Married 1  Don’t wish to answer 9 |
| 6 | Number of children | \|___\|___\| children  Don’t wish to answer 9 |
| 7 | How many deployments have you had? | None 1  One 2  Two 3  Three or more 4  Don’t wish to answer 9 |
| 8 | How well do you agree with the following statements about you? | |
|  | 1. I control my emotions by not expressing them. 2. I tend to focus more on the positive aspects of my life than the negative. 3. It is hard for me to snap back when something bad happens 4. I usually come through difficult times with little trouble | **SD-strongly disagree, D- disagree; N-neutral; A- agree; SA- strongly agree**   \| SD \| D \| N \| A \| SA \| \| --- \| --- \| --- \| --- \| --- \| \| 1 \| 2 \| 3 \| 4 \| 5 \| \| 1 \| 2 \| 3 \| 4 \| 5 \| \| 1 \| 2 \| 3 \| 4 \| 5 \| \| 1 \| 2 \| 3 \| 4 \| 5 \| |
|  |  |  |
|  |  |  |
|  |  |  |
| Section 2: Now, we’d like to know more about your work. Workload and burnout | | |
| 9 | What specialty of healthcare do you provide? | Surgery 1  Pediatrics 2  OBGYN 3  Emergency Medicine 4  Primary Care 5  Psychiatry 6  Other(specify) 8  Don’t wish to answer 9 |
| 10 | What type of health provider are you | Nurse 1  Doctor 2  Physical/occupational therapist 3  Pharmacist 4  Clinical Manager/supervisor 5  Other 8  Don’t wish to answer 9 |
| 11 | How long have you been practicing as a health provider | \|___\|___\| years  Don’t wish to answer 9 |
| 12 | How many hours a week do you work typically? | \|___\|___\| hours  Don’t wish to answer 9 |
| 13 | Which of these currently best describes your workload | Overworked 1  Managing 2  Underworked 3  Don’t wish to answer 9 |
| 14 | Which words best describe the atmosphere in your primary work area? | Calm 1  Busy, but reasonable 2  Hectic, chaotic 3  Something else (specify) 8  Don’t wish to answer 9 |
| 15 | How would you describe your level of compassion for your patients | Always high 1  Always low 2  Sometimes high or low 3  Other(specify) 8  Don’t wish to answer 9 |
| 16 | Which of the following is your workload currently impacting? **Select all that apply** | Physical health A  Mental health B  Patient care C  Job satisfaction D  Personal time E  Family relationships F  Relationship with coworkers G  Quality of life H  Other (specify) X  None of the above Y  Don’t wish to answer Z |
| 17 | Who do you confide in about your struggles at work? **Select all that apply** | Spouse/significant other A  Family member B  Colleague C  Friend outside work D  Other (specify) X  None of the above Y  Don’t wish to answer Z |
| 18 | **How well would you agree with these statements? Tell us whether you strongly disagree, disagree, are neutral, agree or strongly agree** | |
|  | 1. I have the skills and expertise to function in my role 2. My professional values are well aligned with my department leaders’ 3. Overall, I am satisfied with my current job 4. I feel a great deal of stress because of my job: 5. My participation in hobbies/interests have declined because of my work. 6. I have received minimal support at work regarding coping with stress. 7. I am happy with my career path and promotion plan 8. I would apply for this job again 9. I like going to work 10. I can see myself still working here in a year 11. Turnover is a significant problem in my practice overall | **SD-strongly disagree, D- disagree; N-neutral; A- agree; SA- strongly agree**   \| SD \| D \| N \| A \| SA \| \| --- \| --- \| --- \| --- \| --- \| \| 1 \| 2 \| 3 \| 4 \| 5 \| \| 1 \| 2 \| 3 \| 4 \| 5 \| \| 1 \| 2 \| 3 \| 4 \| 5 \| \| 1 \| 2 \| 3 \| 4 \| 5 \| \| 1 \| 2 \| 3 \| 4 \| 5 \| \| 1 \| 2 \| 3 \| 4 \| 5 \| \| 1 \| 2 \| 3 \| 4 \| 5 \| \| 1 \| 2 \| 3 \| 4 \| 5 \| \| 1 \| 2 \| 3 \| 4 \| 5 \| \| 1 \| 2 \| 3 \| 4 \| 5 \| |
|  |  |  |
|  |  |  |
|  |  |  |
|  |  |  |
|  |  |  |
|  |  |  |
|  |  |  |
|  |  |  |
|  |  |  |
| **19** | **Which of the following words describes these statements about your work: poor, marginal, satisfactory, good, optimal?** | |
|  | 1. My control over my workload is: 2. The degree to which my care team works efficiently together is 3. Sufficiency of time for documentation is: 4. My proficiency with EHR use is: | **P-Poor; M- Marginal; S-Satisfactory; G-Good; O-Optimal**   \| P \| M \| S \| G \| O \| \| --- \| --- \| --- \| --- \| --- \| \| 1 \| 2 \| 3 \| 4 \| 5 \| \| 1 \| 2 \| 3 \| 4 \| 5 \| \| 1 \| 2 \| 3 \| 4 \| 5 \| \| 1 \| 2 \| 3 \| 4 \| 5 \| |
|  |  |  |
|  |  |  |
|  |  |  |
| 20 | Which of the following job-related symptoms have you experienced in the past year? **Select all that apply** | Irritability towards co-workers A  Apathy towards patients/job B  Chronic fatigue C  Forgetfulness/impaired concentration D  Anxiety E  Anger F  Depression G  Insomnia H  Increased illness I  Loss of appetite J  Other (specify) X  None of the above Y  Don’t wish to answer Z |
| 21 | Select the one statement that best describes your experience with burnout? | I enjoy my work. I have no symptoms of burnout A  I am under stress, and don’t always have as much energy as I did, but I don’t feel burned out B  I am definitely burning out and have one or more symptoms of burnout C  The symptoms of burnout that I am experiencing won’t go away. I think about work frustrations a lot D  I feel completely burned out. I am at the point where I may need to seek help E |
| 22 | **How well would you agree with these statements? Tell us whether you strongly disagree, disagree, are neutral, agree or strongly agree** | |
|  | - I frequently see burnout in my colleagues - I have felt burnout at my job before - I regularly feel burnout at my job - Burnout has affected my performance at work | **SD-strongly disagree, D- disagree; N-neutral; A- agree; SA- strongly agree**   \| SD \| D \| N \| A \| SA \| \| --- \| --- \| --- \| --- \| --- \| \| 1 \| 2 \| 3 \| 4 \| 5 \| \| 1 \| 2 \| 3 \| 4 \| 5 \| \| 1 \| 2 \| 3 \| 4 \| 5 \| \| 1 \| 2 \| 3 \| 4 \| 5 \| |
| 23 | Which of the following is your burnout currently impacting? **Select all that apply** | Your physical health A  Your mental health B  Patient care C  Job satisfaction D  Personal time E  Family relationships F  Relationship with coworkers G  Quality of life H  I don’t have burnout W  Other (specify) X  None of the above Y  Don’t wish to answer Z |
| 24 | What are the things that stress you about your work? **Select all that apply** | Lack of autonomy A  Difficulty balancing personal and professional life B  Excessive administrative tasks C  High patient volume……… D  Chaotic work environment E  Time pressures F  Compensation/ financial concerns G  Other (specify) X  None of the above Y  Don’t wish to answer Z |
| 25 | What are some ways that you cope with stress/burnout? **Select all that apply** | Taking time off/ vacation A  Time with friends and family B  Exercise C  Stimulants D  Eating E  Yoga or meditation F  Alcohol G  Having another job H  Working part time I  Therapy J  Other (specify) X  None of the above Y  Don’t wish to answer Z |
| 26 | Which of the following does your practice do, to promote well-being? **Select all that apply** | Collect or discuss data related to well-being A  Initiate wellness interventions B  Conduct workshops and education C  Convene social events D  Other (specify) X  None of the above Y  Don’t wish to answer Z |
|  | Section 3: Could you tell us about retention at your practice. | |
| 27 | In the past 12 months, have you thought about leaving your current job as a health provider? | Yes 1  No 2  Don’t wish to answer 9 |
| 28 | What makes you think about leaving your current job as a health provider? **Select all that apply** | Bureaucracy and paperwork A  Workload and stress B  Entering data in EHR C  Not enough compensation/ remuneration D  Not enough time with patients E  Too much time with patients F  I am old enough G  I am not happy with my job H  Other (specify) X  None of the above Y  Don’t wish to answer Z |
| 29 | What could change your mind about leaving your current job as a health provider? | —------------------------------------------------------------------------------------------------------------------------ |
| 30 | How likely are you to remain in the military immediately after your term of service? | Very likely 1  Somewhat likely 2  Somewhat unlikely 3  Very unlikely 8  Don’t wish to answer 9 |
| 31 | What are some of the things that make you think of leaving the military immediately after your term of service? | —----------------------------------------------------------------------------------------------------------------------- |
| 32 | What could change your mind about leaving the military immediately after your term of service? | —------------------------------------------------------------------------------------------------------------------------ |
| 33 | How likely are you to remain in the military till retirement? | Very likely 1  Somewhat likely 2  Somewhat unlikely 3  Very unlikely 8  Don’t wish to answer 9 |
| The study will also be conducting interviews with military providers to humanize the context of burnout among military providers and better understand health provider context and experiences related to burnout. This will help in the design of relevant solutions to address burnout among military providers. | | |
|  | Would you be interested in participating in an interview? | Yes 1  No 2  Don’t wish to answer 9 |
|  | Please include your email address where we can reach you with further information. Your email will not be used for any other purposes. | —--------------------------------------------------------------------------------------------------------------- |
| Thank you so much for your time and willingness to share your experiences | | |
